# Exploring the Intra Entrepreneurship-Employee Engagement-Creativity Linkage and the Diverse Effects of Gender and Marital Status

**DOI:** 10.3389/fpsyg.2021.736914

**Published:** 2021-10-27

**Authors:** Tae-Won Kang, Paresha N. Sinha, Chang-Il Park, Yong-Ki Lee

**Affiliations:** ^1^International Trade and Commerce, College of Social Science, Keimyung University, Daegu, South Korea; ^2^School of Management and Marketing, University of Waikato, Hamilton, New Zealand; ^3^School of Business, Sejong University, Seoul, South Korea

**Keywords:** entrepreneurship, creativity, engagement, corporate social responsibility, SMEs

## Abstract

This research examines which of the sub-dimensions of intra entrepreneurship (innovativeness, pro-activeness, risk-taking), and corporate social responsibility (CSR) support affects employee engagement (organizational and job engagement), which leads to employee creativity. The study uses survey data from SME employees in South Korea and applies the Structural Equation Modeling (SEM)-Artificial Neural Network (ANN) approach, to find that innovativeness and CSR support affect creativity through mediating roles of organizational engagement and job engagement, where job engagement plays a mediating role in the relationship between organizational engagement and creativity. The study also examines how employee gender and marital status effects the relative importance of intra entrepreneurship, organizational engagement, and job engagement on creativity. Findings of ANN analysis evaluates the effects per group (male-unmarried, male-married, female-unmarried, female-married) and shows how the importance of organizational engagement, job engagement, CSR support and innovativeness differ for each group. Contribution to theory and practice are discussed.

## Introduction

In business management, complex problem-solving skills, critical thinking, and creativity are often considered essential competencies for employees (Kruchoski, 2016). However, amid changes in the business environment, the spirit of entrepreneurship in organizations or intra entrepreneurship is considered an important driver of innovative action in terms of exploring new business opportunities and sustaining growth. Intra entrepreneurship (or intrapreneurship) is defined as entrepreneurship within an existing organization, referring to emergent behavioral intentions and behaviors of an organization that are related to departures from the customary (Antoncic and Hisrich, 2003, p. 9). It is the process by which individuals inside organization pursue opportunities irrespective of the resources they control (Stevenson and Jarillo, 1990). Hence, intra entrepreneurship is important for organizational development and economic development in large firms (Guth and Ginsberg, 1990) and SMEs (Covin, 1991; Carrier, 1994). Although the Schumpeter (1934) model presents entrepreneurship and large enterprise as the central axes of economic growth in a country, the effect of scale economies within organizations has become more effective and prominent in small businesses than large enterprises (Kim, 2018). This is explained in part, due to intensifying competition as a result of globalization, as well as the introduction of flexible production methods (Acs and Audretsch, 1993; Kim, 2018). Thus, there is interest in studying intra entrepreneurship in SMEs who are the engines of national growth (Carrier, 1994; Antoncic and Hisrich, 2000, 2001, 2003; Krauss et al., 2005). For example, in South Korea, while there are a number of large enterprises such as Samsung Electronics, Hyundai Motor and LG Electronics; South Korean SMEs play have also played an important role in South Korea's economic growth and development. SMEs account for 99% of Korean companies, 83.0% of employment, and 34.0% of exports (https://www.mss.go.kr/site/eng/02/20201000000002019110604.jsp) (Ministry of SMEs and Startups).

SMEs are different from large companies in terms of their corporate size, organizational structure, ability to cope with the environment, corporate management style, and in particular, their ability to compete with other companies. The competitiveness of SMEs consists of four main points: entrepreneurial competency, internal competency within the company, the external environment, and long-term performance (Man et al., 2002). To improve performance a deliberate internal behavior or entrepreneurial drive is needed that introduces fundamental change in processes, thus allowing for new ideas, creativity, and commitment to be nurtured in the organization (Covin and Miller, 2014). Therefore, the SME owners need to draw creativity from his/her employees, the main resource in the company's internal competencies, and it is necessary to accurately judge and implement these creative ideas. Therefore, apart from the SME owner the behavior and activities of the core workforce, have a strong influence on its competitiveness (Man et al., 2002).

Research has suggested firms that nurture organizational structures and values conducive to intra entrepreneurial activities, and which have entrepreneurial orientations that enhance innovation, risk taking and proactive responses, usually report higher growth and profitability than organizations that are lacking such characteristics (Antoncic, 2007). The concept of intra entrepreneurship is useful to differentiate between conservative and more entrepreneurial firms (Covin, 1991; Antoncic and Hisrich, 2003) where innovativeness, proactiveness, and risk-taking are the main characteristics of organizational level entrepreneurship (Covin and Slevin, 1991). This suggests entrepreneurial attitudes and behavior of the SME owner and employees can explain business growth, revitalization, and company's overall success (Zahra et al., 2000; Kuratko et al., 2005). In addition, emphasizing innovative, social value creating activity and achieving social objectives through the display of innovativeness, proactiveness, and risk taking can help the firm seek opportunities for growth from social impact (Austin et al., 2006; Weerawardena and Mort, 2006; Kreiser et al., 2013). Supporting social responsibility activities can also fulfill employees' personal needs for meaningfulness and value building (Austin et al., 2006). Therefore, this study of intra entrepreneurship encompasses organizational level factors (orientation/support for commercial and social entrepreneurship in the organization) and individual level outcomes (employee engagement and creativity), as employees' perceptions of organization's support for entrepreneurial activity can influence their cognitions and drive their motivational levels to act in an innovative way (Urban and Wood, 2017; Neessen et al., 2019).

Previous research has suggested there are gender differences related to entrepreneurship and intra entrepreneurship behaviors, due to psychological factors such as women's emotional response to uncertain situations, overconfidence of men, and how men and women perceive and act in risky situations (Croson and Gneezy, 2009; Adachi and Hisada, 2017). Research has suggested, due to positive change in their personal resources, entrepreneurship fosters work engagement (Gawke et al., 2017), which is one of the main strategic drivers for performance, growth, and sustainable competitive advantage (Kassa and Raju, 2015). However, how gender and marital status of employees effects entrepreneurship- work engagement is not explored in the SME context. Based on this discussion, in this study we examine gender differences in terms of how employees experience support for intra entrepreneurship activities and how that influences employee engagement. In addition, based on a person-environment fit (P-E fit theory) (Kristof, 1996; Shalley et al., 2004), we propose that employee organizational and job engagement be viewed as critical constructs that increase employee's creativity.

Our overarching RQ is: Which of the sub-dimensions of entrepreneurship (including social entrepreneurship support) influences employee engagement and, what is its impact on employee creativity. The intra entrepreneurship-organizational and job engagement-creativity dynamic suggested in this study are examined using SEM analysis. However, the SEM analysis can have different results depending on employees' gender and marital status. Therefore, study is designed to use a SEM-ANN approach, which combines SEM analysis, which explains the linear (compensatory) relationship, and ANN analysis, which explains the non-linear (non-compensatory) relationship to explain the combination of influence or criteria variables. The effects of the sub-dimension of entrepreneurship, organizational and job engagement, and creativity according to gender (male, female) and marital status (unmarried, married) are examined through an artificial neural network (ANN) analysis (Scott and Walczak, 2009). This is useful in that effects can be evaluated per group (male-unmarried, male-married, female-unmarried, female-married).

The current study contributes in at least three ways to the literature on entrepreneurship, employee engagement, and creativity. First, it advances our understanding of an expanded concept of the intra entrepreneurship—by including the support for CSR dimension into the sub-dimensions of entrepreneurship and advances our understanding of support for intra entrepreneurship- engagement link for SME employees. Second, by measuring two types of employee engagement namely organizational engagement and job engagement, we are better able to identify if the causal relationship between intra entrepreneurship and employee engagement differs for male and female employees. Third, our research suggests that creativity indicators should be used as indicators to explain sustainable competitive advantage and growth in rapidly changing business environments by using creativity as a performance indicator of the effectiveness of entrepreneurship. Fourth, using gender socialization processes (Eddleston and Powell, 2008) and social role theory (Eagly, 1987), this research examines the roles of gender and marital status as moderators in the relationship between entrepreneurship, employee engagement, and creativity. This study aims to help management understand that the relationship between intra entrepreneurship-employee engagement-creativity differs according to the individual characteristics of employees, enriching the understanding of employee management using entrepreneurship.

The rest of the study is structured as follows: first, the literature review and hypothesis development are presented. Second, the methodology is presented. Third, the empirical findings are presented. Finally, the theoretical and managerial implications are discussed.

## Literature Review and Hypothesis Development

### Intra Entrepreneurship in SMEs

Entrepreneurship focuses on novelty in the form of new products or services, new administrative processes, and entry to new product or geographical markets as the drivers of wealth creation (Lumpkin and Dess, 1996; Antoncic and Hisrich, 2003; Ireland et al., 2003). According to Resource based theory (RBT), the domain of entrepreneurship is about cognition, discovery, pursuing market opportunities, and coordinating knowledge that lead to heterogeneous outputs (Alvarez and Busenitz, 2001, p.757).

The term intra entrepreneurship or entrepreneurship in organizations is used to explain the creation of solutions for challenges facing the firm, the development of new products, services or new ways of dealing with existing or new customers (Antoncic and Hisrich, 2001, 2003). Building on Miller (1983), previous research has suggested risk-taking, progressiveness, and innovation-driven organizational activities, as generally influencing business performance and competitive advantage (Miller, 1983; Lumpkin and Dess, 1996; Weerawardena and Mort, 2006; Linton and Kask, 2017) including in the case of SMEs (Rhee et al., 2010; Cui et al., 2018; Genc et al., 2019). Intra entrepreneurial activities of employees that include initiation, risk taking, and ideation or coming up with new ideas (Jong et al., 2015) can help organization to adapt to internal and external threats and proactively act upon emerging opportunities and employees should be motivated to do so (Ireland et al., 2003). Intra entrepreneurship can be classified into four dimensions: (1) new business venturing, (2) innovativeness, (3) self-renewal, and (4) proactiveness (Antoncic and Hisrich, 2001). New business venturing refers to the creation of new businesses within the existing organization regardless of the level of autonomy of the organizational decision maker, whereas innovativeness focuses on the product and service innovation with emphasis on the use or development of technology or demonstrating technological leadership (Covin and Slevin, 1991; Antoncic and Hisrich, 2001). The self-renewal dimension refers to organizational change, and the firm's capability to adapt and remain flexible and change the way in which it competes (Antoncic and Hisrich, 2001; Fitzsimmons et al., 2005).

Therefore, it is necessary to re-clarify the dimensional structure of intra entrepreneurship concept by bounding it at the organizational level of analysis (Antoncic and Hisrich, 2003). Building on Miller (1983) researchers have conceptualized organizational level entrepreneurship or Entrepreneurial Orientation into three sub dimensions (innovativeness, pro-activeness and risk taking) or as a five dimensional construct (Zellweger and Sieger, 2012; Wales et al., 2013; Covin and Wales, 2019) and applied it in the context of SMEs (Isichei et al., 2020). The other two sub dimensions added by Lumpkin and Dess (1996) are competitive aggressiveness and autonomy. We explain the various sub dimensions, while highlighting that the five-dimensional conceptualization has not been widely adopted (Wales et al., 2013).

Innovativeness is understood as the tendency to promote a company's experiments, new ideas, and creative processes, as well as to support the creation of new products, services, and processes (Lumpkin and Dess, 1996) and can be studied in terms of outcome or process (Linton, 2019). In addition, entrepreneurial innovativeness is a company's effort to continue to work and change organization for the purpose of finding new opportunities and solutions despite the uncertainty and limited resources of the external environment (Miller, 1983). Higher level of innovativeness supports creativity and experimentation in the organization (Lumpkin and Dess, 1996).

Pro-activeness refers to a company's tendency to actively anticipate and exercise future opportunities and market demands, which means that a company has the ability to preoccupy the market and have market cultivation power (Lumpkin and Dess, 1996). Pro-activeness reflects the willingness to introduce new products or to boldly participate in the market, create new products or services before competitors, and shape change in the environment to create future demand (Keh et al., 2007) and can be studied in terms of process or outcome (Linton, 2019).

Risk-taking is defined as the degree of willingness to boldly challenge things, even if the results are uncertain (Sexton and Bowman, 1986) and can be studied as outcome or process (Linton, 2019). Risk-taking refers to managers' willingness to pursue opportunities using their own resources without being bound by any risk or environment to capture new opportunities. Risk-taking at the organizational level is described as an adventurous entry into a new market or taking a large risk with uncertain outcomes (Covin and Slevin, 1991).

Competitive aggressiveness, which refers to how firms react to competitive trends that already exist in the marketplace. As this sub-dimension of entrepreneurship is less about creating new solutions, and potentially more relevant for larger firms in mature industries (Lumpkin and Dess, 2001), is becomes less important for this study on intra entrepreneurship in SMEs. The sub dimension autonomy refers to owners valuing their own decision-making, which is more relevant for SME owners and has lower importance for managers or employees (Krauss et al., 2005).

### Social Entrepreneurship and Support for CSR in SMEs

In recent years, the scope of entrepreneurship has expanded to place corporate existence in symbiosis with sharing in society (Austin et al., 2006). Accordingly, the concept of social entrepreneurship has recently become more imperative (Austin et al., 2006; Kreiser et al., 2013), and there is interest in entrepreneurship that strives to achieve social value creation through the display of innovativeness, proactiveness, and risk taking (Weerawardena and Mort, 2006). CSR refers to corporate activities that aim to realize and recognize the importance of social and economic responsibility in the decision-making process of corporate management (Anderson et al., 2007). There are similarities between outcomes of commercial and social entrepreneurship practiced *via* CSR. CSR can be a factor that maximizes corporate profits in the long run (Baron, 2007) and it can provide rewards such as growth, which leads to sustainability, therefore SMEs need to approach support for CSR strategically (Stoian and Gilman, 2017). Previous research suggests support for corporate social responsibility influences employee attitudes and behavior (Tian and Robertson, 2019) and SMEs also expect substantial benefits from visible CSR activities, such as support from stakeholders (Tilley, 2000). Compared to large companies SMEs are embedded in the local communities, maintain a close relationship with many stakeholders, and, most decisions are made by the owners (Demuijnck and Ngnodjom, 2013; Choongo, 2017). Therefore, CSR support activities of SMEs owner will affect not only profits (Nguyen and Nguyen, 2021), but also the attitudes and behaviors of employees toward the organization and job (Abdelmotaleb et al., 2018).

### Employee Engagement (Organizational and Job Engagement)

Support for intra entrepreneurship can foster an employee's agentic behaviors, that increase work engagement (Gawke et al., 2017), which is one of the main strategic drivers for performance, growth, and sustainable competitive advantage (Kassa and Raju, 2015). Employee engagement is a voluntary physical-cognitive-emotional energy for the active performance of an individual that is evident when the basic psychological needs of the individual are satisfied (Kahn, 1990). Employee engagement theory found in Kahn (1990) suggests that a person's degree of engagement in their work role is a function of three different psychological conditions. First, their experience of psychological meaningfulness or feeling valued; second, presence of psychological safety as it allows the individual to express their ideas without negative consequences to their status or career; and third, their psychological availability or belief that they possess the psychological, emotional and physical resources to invest into their on the job performance (Saks and Gruman, 2014). CEOs prefer enthusiastic employees because they are more likely to be devoted to fulfilling their roles and contributing to the organization with high job and organizational performance (Xanthopoulou et al., 2009; Rich et al., 2010).

Saks (2006) first conceptualized the concept of employee engagement as a theory of engagement based on social exchange theory (SET), dividing it into organizational and job engagement. Organizational engagement is defined as an individual's psychological role in an organization by doing his/her best as a member of the organization. In addition, it means that when employees are provided with abundant resources from the organization, the employees themselves have an obligation to reciprocate this behavior, thereby increasing their degree of engagement in the organization (Kahn, 1990).

Meanwhile, Saks (2006) conceptualized job engagement based on the Maslach et al.'s (2001) model, which defined job engagement as the degree to which an individual pays attention to the performance of his/her role and absorbs his/her work. Job engagement is therefore more than the belief of employees (Schaufeli and Bakker, 2010), as it incorporates an individual's enthusiasm toward their job. Engaged employees are absorbed in their work, open to new experiences and are willing to acquire new skills to be creative in their effort to improve their performance (Bakker and Xanthopoulou, 2013).

### Creativity

Amabile et al. (1996) define creativity as “the production of novel and useful ideas in any domain” (p. 1155). Hence, employee creativity is needed to bring new ideas and provide better solutions to existing problems and increase the firm's competitive advantage (Hirst et al., 2009). When defining entrepreneurship, the link between creativity and innovation is often emphasized. Creativity as a pre-condition of innovation (West and Farr, 1990), needed for changing or creating products, services, and processes helping to achieve organizational goals (Oldham and Cummings, 1996). Taylor's (1960) definition focuses on new products and creative thinking, whereas Stevenson and Gumpert (1985) emphasized the relationship between entrepreneurship and creativity as entrepreneurial behavior that promotes creativity and flexibility. In other words, in entrepreneurial organizations the practices, values are supportive to the creativity of the individual organization members (Bakker and Xanthopoulou, 2013).

### Theoretical Framework and Hypothesis Development

Based the above discussion, this study proposes a framework for testing the relationship between intra entrepreneurship—employee engagement—creativity. Building on resource-based theory (RBT), it considers intra entrepreneurship as the internal resources or capabilities of a company (Alvarez and Busenitz, 2001) and the social responsibility of SMEs is represented as CSR support a factor constituting the social entrepreneurship of SMEs (Buendía-Martínez and Carrasco Monteagudo, 2020). Building on employee engagement theory (Kahn, 1990; Saks, 2006; Rich et al., 2010), it considers why SME owners support for intra entrepreneurship will affect employees organizational and work engagement. The framework focuses on individual employee creativity from the perspective of a person-environment fit (P-E fit) theory (Kristof, 1996; Shalley et al., 2004), which emphasizes the importance of interaction between individuals and their work-related environment. Whereas, entrepreneurship affects business performance, SMEs owners are concerned with the impact of their entrepreneurship on employee attitudes and outcomes such as organizational and job engagement, and creativity. In this framework as entrepreneurship influences how employees shape their attitudes toward organizations and jobs, SMEs owners should use intra entrepreneurship and support for CSR as a resource to motivate their employees to remain engaged on their jobs and improve their creativity. On the other hand, the attitudes and reactions of these employees will differ according to their gender and marital status.

These differences can arise from entrepreneurship intentions which are found to be higher in men than in women (Zhao et al., 2005; De Tienne and Chandler, 2007; Gupta et al., 2009; Nowiński et al., 2017). Men experience better outcomes for their ideas due to unconscious gender bias of evaluators (Yang et al., 2020). In particular, entrepreneurship is significantly lower in women who give birth (Choo and Kong, 2019), although the effect of entrepreneurship education is higher in women than men (Oosterbeek et al., 2010, Westhead and Solesvik, 2016). Therefore, there may be gender differences in each sub-dimension of intra entrepreneurship as a result of factors including women's socialization process and explained by social role theory.

Social role theory is found upon the belief that human beings have inherent behavioral differences in everyday life (Eagly, 1987), stemming from cultural and social stages (McWhirter, 1997; Lent et al., 2000) as well as primary traditional gender characteristics (Eagly, 1997). In addition, the process of socializing women is not only a process for people to learn to work in a particular organization, but also to lead them to accept and believe in the behavioral conventions within an organization (Taormina, 1994, 1997, 2004).

One of the essential differences between unmarried and married women is family responsibility (Deligero and Laguador, 2014). In general, women tend to experience career breaks due to social constraints, cultural values, and environmental factors (McWhirter, 1997; Lent et al., 2000), and more men are married than not. As such, there is a tendency amongst married women to increase responsibility for the family and decrease engagement at work.

**Figure 1:**
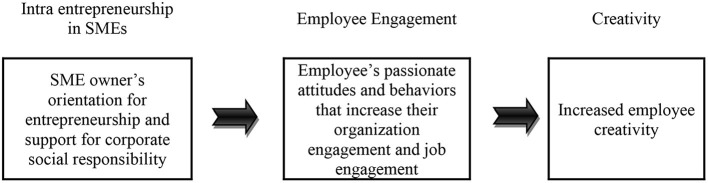
Theoretical framework.

Figure 1 illustrates this general theoretical framework; however, the importance of organizational engagement, job engagement, CSR support and innovativeness is likely to differ for married/unmarried male and female employees. Below, specific generalizations are suggested with respect to outcomes of organizational and job engagement and then with creativity.

Intra entrepreneurship is explained by three basic desirable components: innovativeness, pro-activeness, and risk-taking. These components have been suggested by researchers as generally influencing business performance and competitive advantage (Miller, 1983; Lumpkin and Dess, 1996; Weerawardena and Mort, 2006) as well as in the case of SMEs (Rhee et al., 2010; Cui et al., 2018; Genc et al., 2019). Previous studies found that that there is a positive relationship between intra entrepreneurship and work engagement, especially when employees have psychological capital (Pandey et al., 2021). In particular, employees can achieve social value creation through the display of innovativeness, proactiveness and risk-taking (Weerawardena and Mort, 2006) when they invest their emotional and cognitive energies into their work (Bakker et al., 2011; Pandey et al., 2021).

In terms of the causal relationship between organizational and job engagement, organizational engagement has been noted as a stronger leading factor in outcome variables such as job satisfaction, organizational commitment, and organizational citizenship compared to job engagement, when employees perceive that they receive support from an organization repeatedly (Saks, 2006). Organizational engagement delivers the shared values of the organization to employees and provides a motivational environment in which to work (Barrick et al., 2015). The fact that organizational engagement is an antecedent of job engagement means that if organizational engagement is increased by intra entrepreneurship, job engagement can be increased, which can improve the individual's ability and performance, resulting in enhanced employee creativity. For example, Kassa and Raju (2015) claimed that entrepreneurship plays a role in creating an organizational culture for fostering creativity and innovation. Other studies found that perceived organizational CSR initiatives by employees increase employees' creativity (Hur et al., 2018; Chaudhary and Akhouri, 2019). Social and sustainable dimensions of CSR most strongly predicted social and affective dimensions of employee engagement (Duthler and Danish, 2018). CSR increases employee engagement by allowing employees to do more work (Glavas, 2016) and increases employees' group identification (Hameed et al., 2016). Therefore, integration of CSR into the core of a company, both socially and in terms of the organizational support of the company, is fundamental for success. Some studies report that SMEs' CSR activities influence employees' organizational and job attitudes (Abdelmotaleb et al., 2018) and performance (Hur et al., 2018; Chaudhary and Akhouri, 2019). Therefore, we propose following hypotheses:

**H1:** Intra entrepreneurship has a positive effect on organizational engagement.
**H1-1:** Innovativeness has a positive effect on organizational engagement.
**H1-2:** Pro-activeness has a positive effect on organizational engagement.
**H1-3:** Risk-taking has a positive effect on organizational engagement.
**H1-4:** CSR support has a positive effect on organizational engagement.
**H2:** Intra entrepreneurship has a positive effect on job engagement.
**H2-1:** Innovativeness has a positive effect on job engagement.
**H2-2:** Pro-activeness has a positive effect on job engagement.
**H2-3:** Risk-taking has a positive effect on job engagement.
**H2-4:** CSR support has a positive effect on job engagement.
**H3:** Organizational engagement has a positive effect on job engagement.

Employee engagement plays a critical role in developing employee creativity (e.g., Eldor and Harpaz, 2016; Asif et al., 2019; Ismail et al., 2019). This means that how and when SME owners create an organizational environment that stimulates and supports employees' creative work involvement as a social process is an important challenge (Bouckenooghe and Menguç, 2018). From this perspective, intra entrepreneurship, and organizational engagement at the level of organization and job engagement at the level of individual could facilitate employee creativity. Therefore, we propose following hypotheses.

**H4:** Organizational engagement has a positive effect on creativity.
**H5:** Job engagement has a positive effect on creativity.

## Methodology

### Data Collection

Our sample consists of employees from SMEs in South Korea. According to article 3 of the Enforcement Decree of the Framework Act on SMEs, total assets of an SMEs shall not exceed KRW 500 billion regardless of business type. Also, the maximum average sales of SMEs should be less than KRW 150 billion, not the number of employees https://www.mss.go.kr/site/eng/02/20201000000002019110604.jsp. Firms were randomly selected from the KODIT (Korea Credit Guarantee Fund) database. Korea Credit Guarantee Fund (KODIT) is a public financial institution established in 1976 under the provisions of the Korea Credit Guarantee Fund Act (www.kodit.co.kr). We used stratified convenience sampling method to obtain the data from respondents who were employees of SMEs in different industries, selected based on the percentage of industry composition of the population of firms in South Korea. The purpose of the study was explained to employees working at participating companies, and only those who agreed to participate in the study were sent the survey questionnaire employees (https://www.mss.go.kr/site/eng/02/20201000000002019110604.jsp) (Ministry of SMEs and Startups). In addition, the participants were informed that the confidentiality of the information collected was assured. In order to increase response rate, a small gift was offered to all participants. Four hundred copies of the questionnaires were distributed, and 370 responses were returned. A total of 285 responses were used for the empirical analysis, after discarding 27 copies that were not SMEs and 58 questionnaires with incomplete or insufficient responses. The data were analyzed using SPSS Win 22.0 and SmartPLS 3.3.3 program.

### Measures

All measurement items used in this study were measured using a 7-point Likert scale, from 1 point = “strongly disagree” to 7 points = “strongly agree.” Intra entrepreneurship was classified into four sub-dimensions, innovativeness (4 items) (e.g., The owner of our company is interested in the development and improvement of new menus, products, and services.), pro-activeness (4 items) (e.g., The owner of our company tends to actively respond to and act on environmental changes.), risk-taking (4 items) (e.g., The owner of our company tries to actively enter new business areas even in uncertain situation.). These items were based on the literature (Miller, 1983; Covin and Slevin, 1986; Barringer and Bluedorn, 1999), and CSR support was measured using 6 items. (e.g., The owner of our company is active in environmental protection activities.). These items were based on (Lee et al., 2016). Employee engagement was classified into two sub-dimensions, organizational (6 items) (e.g., Being a member of this company is very captivating.) and job engagement (8 items) (e.g., I really “throw” myself into my job.) based on previous studies (Saks, 2006; Lee et al., 2014). Creativity was measured using 4 items (Wu et al., 2008) (e.g., I tend to try new ideas or methods.). In addition, gender was measured by two nominal variables of female (0) and male (1), and marital status was measured using the nominal scale of unmarried (0) and married (1).

## Results

### Demographic Profiles of Respondents

According to the demographic status of respondents (employees) (see Table 1), the ratio of males (70.2%) to females (29.8%) was about 7 to 3, and the ratio of married (58.9%) to unmarried (41.1%) was about 6–4. The main age group was employees in their 30s (42.1%), and their education was mostly college and university level (78.3%). The working period was either <3 years (46.7%) or between 3 and 10 years (35.4%). Responsibilities are in the order of sales and marketing (29.1%) and planning and general affairs (26.0%). Respondents were distributed evenly in the order of chief (24.2%), deputy director and director (25.3%), or manager (22.5%). Average monthly income was <2–3 million won (35.8%) or <3–4 million won (25.6%).

**Table 1:** Socio-demographic profiles of respondents (*n* = 285).

		***n***	**%**
Gender	Male	200	70.2
	Female	85	29.8
Marital status	Unmarried	117	41.1
	Married	168	58.9
Age	20–29	56	19.7
	30–39	120	42.1
	40–49	73	25.6
	Over 50	36	12.6
Education	Below high school	30	10.5
	Junior college	68	23.9
	University	155	54.4
	Graduate school	32	11.2
Monthly income (Million won)	1-less than 2	52	18.2
	2-less than 3	102	35.8
	3-less than 4	73	25.6
	4-less than 5	22	7.7
	5-less than 10	30	10.5
	More than 10	6	2.2
Working period (year)	Under 1	57	20.0
	1-under 3	76	26.7
	3-under 5	37	13.0
	5-under 7	32	11.2
	7-under 10	32	11.2
	Over 10	51	17.9
Type of tasks	Products	15	5.3
	Sales/marketing	83	29.1
	Planning/general affairs	74	26.0
	R&D	12	4.2
	Technology	35	12.3
	Others	66	23.1
Position	Staff	49	17.1
	Chief	69	24.2
	Manager	64	22.5
	Deputy director/director	72	25.3
	Executive	31	10.9
Type of company	Venture	57	20.0
	Innobiz	19	6.7
	Innovative company	26	9.1
	General company	169	59.3
	Others	14	4.9
Number of employees	Less than 10	50	17.5
	10-less than 20	74	26.0
	20-less than 30	29	10.2
	30-less than 50	35	12.3
	50-less than 100	28	9.8
	100-less than 300	42	14.7
	300-less than 500	27	9.5
Sectors	Manufacturing	107	37.5
	Distribution (wholesales, retailing)	53	18.6
	Information (IT)	28	9.8
	Service	80	28.1
	Others	17	6.0

### Assessing Validity and Reliability

Confirmatory factor analysis (CFA) was used to test how well-measured variables represent a smaller number of constructs with SmartPLS 3.3.3, because we choose variance-based structural equation modeling using partial least squares (PLS-SEM) (Hair et al., 2017). To assess the convergent validity, the measures are factor loading and Average variance extracted, while HTMT was used to assess discriminant validity. As shown in Table 2, the levels of internal consistency reliability were established because the values of factor loadings, Cronbach's α and composite reliabilities (CR) were larger than 0.7, and convergent validity was confirmed because the values of average variance extracted (AVE) were greater than the acceptable threshold of 0.5, so convergent validity is confirmed. Finally, the heterotrait-monotrait (HTMT) ratio of correlations (Henseler et al., 2015) values, ranging from 0.250 to 0.879, were under 0.900 (see Table 3). Thus, discriminant validity is well-established.

**Table 2:** Measurement model.

**Constructs and items**	**Factor loadings**	**α**	**CR**	**AVE**
*Innovativeness*		0.895	0.935	0.827
The owner of our company is interested in the development and improvement of new menus, products, and services.	0.865			
The owner of our company is flexible in applying new processes.	0.938			
The owner of our company tends to actively embrace and encourage new and innovative ideas.	0.924			
The owner of our company believes that innovation must be promoted to improve competitiveness.	–			
*Pro-activeness*		0.927	0.953	0.872
The owner of our company tends to actively respond to and act on environmental changes.	0.934			
The owner of our company predicts and exploits new opportunities and encourages active and progressive action.	0.940			
The owner of our company tends to find new opportunities and secure leadership positions.	0.928			
The owner of our company tends to encourage passionate and confident participation in job performance.	–			
*Risk-taking*		0.901	0.938	0.835
The owner of our company tries to actively enter new business areas even in uncertain situations.	0.898			
The owner of our company actively and boldly pursue risks to achieve organizational goals and achievements.	0.943			
The owner of our company promotes projects that has the opportunity to raise expected profits despite risks.	0.900			
*CSR support*		0.930	0.945	0.740
The owner of our company is active in environmental protection activities.	0.866			
The owner of our company is immersed in improving the welfare of the community.	0.855			
The owner of our company tends to make donations to solve social problems.	0.820			
The owner of our company prioritizes the interests of customers (including employees and business partners)	0.896			
The owner of our company tries to comply with the relevant laws.	0.854			
The owner of our company tries to comply with ethical standards.	0.870			
*Organizational engagement*		0.964	0.972	0.876
Being a member of this company is very captivating.	–			
I am really into the “goings-on” in this company.	0.908			
Being a member of this company makes me come “alive”	0.948			
Being a member of this company is exhilarating for me.	0.940			
I am really engaged in my work for this company.	0.945			
I am committed to this company.	0.938			
*Job engagement*		0.921	0.938	0.718
I really “throw” myself into my job.	0.882			
Sometimes, I am so into my job that I lose track of time.	0.804			
My mind never wanders and I do not think of other things when doing my job.	–			
I am highly engaged in this job.	0.843			
The job I have makes me enthusiastic.	0.884			
I view my job as being meaningful.	0.794			
I am enthusiastic about the job I do.	0.871			
*Creativity*		0.911	0.938	0.790
I tend to try new ideas or methods.	0.898			
I tend to find new ideas and ways to solve problems.	0.903			
I want to create breakthrough ideas in my field.	0.893			
I can be called a role model of creativity.	0.860			

**Table 3:** Heterotrait-Monotrait ratio (HTMT).

	**1**	**2**	**3**	**4**	**5**	**6**	**7**
1. Innovativeness							
2. Pro-activeness	0.879						
3. Risk-taking	0.620	0.652					
4. CSR support	0.633	0.678	0.587				
5. Organizational engagement	0.606	0.590	0.473	0.744			
6 Job engagement	0.453	0.465	0.365	0.563	0.703		
7. Creativity	0.323	0.337	0.250	0.478	0.538	0.751	

### Common Method Bias Assessment

To reduce common method bias (CMB), procedural and statistical approaches (Podsakoff et al., 2012) were used. In terms of procedural approach, first, we informed participant the study's research purpose and provided them instructions to increase the probability of response accuracy (Podsakoff et al., 2003, 2012). Second, we removed ambiguous items that were difficult for respondents to understand or interpret through a pre-test of the questionnaire. Third, when designing the questionnaire, we changed the order of independent and dependent variables and did not measure continuously in the order presented in the proposed model and performed physical separation to answer online questionnaires on a separate page. In terms of statistical approach, we followed Kock' procedure which assesses CMB using the VIF (variance inflation factor) value (Kock, 2015). The VIF value is lower than 3.3 (VIF = 1.549–2.140), hence CMB is not a problem in this study.

### Assessment of the Structural Model

**Figure 2:**
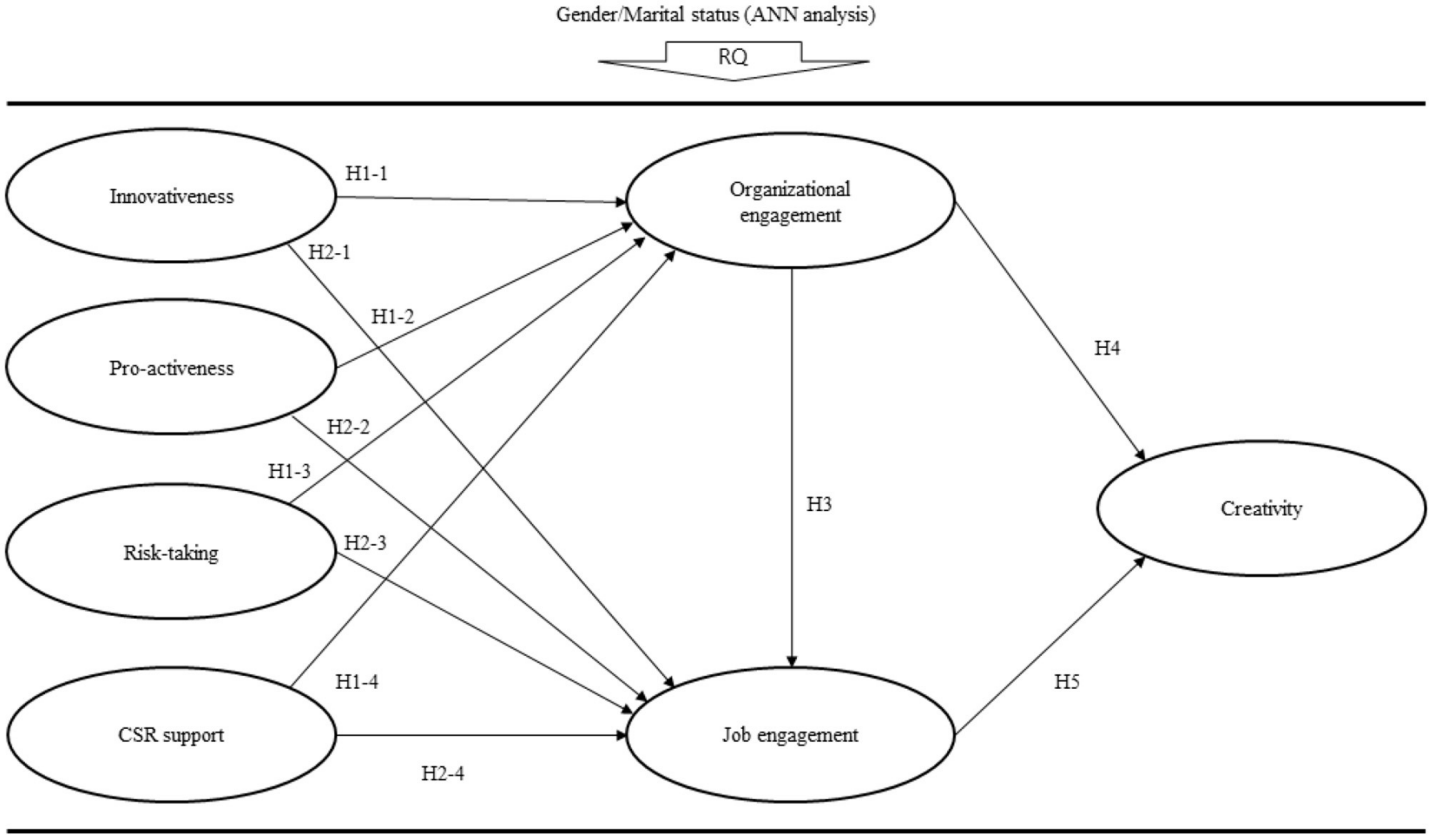
Proposed model.

The proposed model (see Figure 2) was assessed with SmartPLS 3.3.3. PLS is a method of maximizing the explanatory power of endogenous variables. That is, explanatory power and predictive fit were evaluated using analytical methods suitable for research to maximize variance and minimize structural errors (Chin, 1998; Tenenhaus et al., 2005; Hair et al., 2017; Kim et al., 2019; Kim, 2021). First, the variance inflation factor (VIF) was 1.549–2.140, which is <5, therefore indicating that there was no multicollinearity problem. Second, the values of R^2^ for organizational engagement, job engagement, and creativity, which represent the explanatory power of endogenous variables, were as 0.540 (54.0%), 0.449 (44.9%), 0.480 (48.0%), respectively. Hence, the model's predictive powers were acceptable because the values of R^2^ were larger than 0.1 (10%) (Falk and Miller, 1992). Chin (1998) suggested the criteria for explanation power was 0.67 (strong), 0.33 (medium), and 0.19 (weak). Third, the values of the cross-validated redundancy (Q^2^) for organizational engagement (0.467), job engagement (0.314), and creativity (0.373) were larger than 0, indicating that the capabilities of the model were acceptable. Finally, the root mean square residual (SRMR) was 0.047, which was smaller than the reference value of 1 or 0.08, indicating that the predictive power of the model was good.

### Hypotheses Testing

H1-1-1-3 state entrepreneurship influence organizational engagement. Table 4 shows that innovativeness/Pro-activeness (β = 0.362, t = 5.928, *p* < 0.01) and CSR support (β = 0.426, t = 8.010, *p* < 0.01) influence organizational engagement, however, risk-taking does not. Therefore, H1-1 and H1-3 are supported, but H1-2 is not supported. H2-1-2-3 address that entrepreneurship influences job engagement. Contrary to expectations, innovativeness/Pro-activeness (β = 0.056, t = 0.787, n.s.), risk-taking (β = −0.02, t = 0.020, n.s.), and CSR support (β = −0.072, t = 0.977, n.s.) do not influence job engagement, hence results not supporting H2-1-2-3. H3-4 posit that organizational engagement influence job engagement and creativity. As expected, organizational engagement influences job engagement (β = 0.426, t = 8.141, *p* < 0.01). However, organizational engagement does not influence creativity (β = 0.071, t = 1.046, n.s.). Finally, job engagement influences creativity (β = 0.643, t = 12.658, *p* < 0.01), supporting H5.

**Table 4:** Structural estimates (PLS).

	**Paths**	**Estimate**	**f^2^**	**t**	***p***	**Results**
H1-1	Innovativeness → Organizational engagement	0.202	0.031	2.524	0.012	Supported
H1-2	Pro-activeness → Organizational engagement	0.031	0.001	0.387	0.699	Not Supported
H1-3	Risk-taking → Organizational engagement	0.004	0.000	0.060	0.952	Not Supported
H1-4	CSR support → Organizational engagement	0.571	0.387	11.055	0.000	Supported
H2-1	Innovativeness → Job engagement	−0.011	0.000	0.112	0.911	Not Supported
H2-2	Pro-activeness → Job engagement	0.065	0.002	0.629	0.529	Not Supported
H2-3	Risk-taking → Job engagement	0.007	0.000	0.090	0.928	Not Supported
H2-4	CSR support → Job engagement	0.073	0.004	0.882	0.378	Not Supported
H3	Organizational engagement → Job engagement	0.580	0.281	7.767	0.000	Supported
H4	Organizational engagement → Creativity	0.071	0.007	1.245	0.213	Not Supported
H5	Job engagement → Creativity	0.635	0.432	12.663	0.000	Supported
		**R** ^2^	**Q** ^2^			
	Organizational engagement	0.540	0.467			
	Job engagement	0.449	0.314			
	Creativity	0.480	0.373			

### Effect Size (f^2^) Analysis

The relative contribution of exogenous constructs to endogenous construct was assessed using the effect size (f^2^). The three criteria suggested by Cohen (1988) were used; 0.02 (small), 0.15 (medium), and 0.35 (large). As shown in Table 4, the effect size of CSR support (0.235) was medium, and innovation/pro-activeness (0.140) on organizational engagement was small. And the effect size of organizational engagement on job engagement was medium (0.309). Finally, the effect size of job engagement on creativity was large (0.443).

### Mediating Test of Employee Engagement

Mediating roles of organizational and job engagement were tested using bootstrapping (Zhao et al., 2010; Kim et al., 2019). Table 5 shows organizational engagement plays a full mediating role in the relationship between innovativeness and job engagement, because the direct effect of innovativeness on job engagement is insignificant (β = −0.011, t = 0.012, n.s), but the direct effect of innovativeness on organizational engagement is significant (β = 0.202, t = 2.524, *p* < 0.05) and the direct effect of organizational engagement on job engagement is significant (β = 0.580, t = 7.767, *p* < 0.01). Also, organizational engagement plays a full mediating role in the relationship between CSR support and job engagement because the direct effect of CSR support on job engagement is insignificant (β = 0.073, t = 0.882, n.s), but the direct effect of CSR support on organizational engagement is significant (β = 0.571, t = 11.055, *p* < 0.01) and the direct effect of organizational engagement on job engagement is significant (β = 0.580, t = 7.767, *p* < 0.01). In addition, job engagement plays a full mediating role in the relationship between organizational engagement and creativity because the direct effect of organizational engagement on creativity is insignificant (β = 0.083, t = 1.245, n.s), but the direct effect of organizational engagement on job engagement is significant (β = 0.580, t = 7.767, *p* < 0.01) and the direct effect of job engagement on creativity is also significant (β = 0.635, t = 12.663, *p* < 0.01). In addition, Table 5 confirms mediation effects of organizational and job engagement because there is no zero between the lower limit confidence interval (LLCI) and the upper limit confidence interval (ULCI) of confidence interval's (CI).

**Table 5:** Mediating role of employee engagement using bootstrapping.

	**Direct effects** **β** **(t)**			**Indirect effects** **β** **(t)**		
**Paths of mediating role**	**(X → M)**	**(M → Y)**	**(X → Y)**	**(X → M → Y)**	**CI [LLCI, ULCI]**	**Mediating roles**
Innovativeness (X) → Organizational engagement (M) → Job engagement (Y)	0.202 (2.524)^*^	0.580 (7.767)^**^	−0.011 (0.012)^n.s^	0.074 (2.373)^*^	[0.019, 0.142]	Full
CSR support (X) → Organizational engagement (M) → Job engagement (Y)	0.571 (11.055)^**^	0.580 (7.767)^**^	0.073 (0.882)^n.s^	0.331 (6.343)^**^	[0.237, 0.441]	Full
Organizational engagement (X) → Job engagement (M) → Creativity (Y)	0.580 (7.767)^**^	0.635 (12.663)^**^	0.083 (1.245)^n.s^	0.368 (6.321)^**^	[0.264, 0.493]	Full

** *p < 0.01*,

* *p < 0.05, n.s., not significant*.

*LLCI, the lower limit confidence interval*.

*ULCI, the upper limit confidence interval*.

### ANN Analysis

In order to identify the importance of intra entrepreneurship, organizational, and job engagement on creativity, sensitivity analysis was conducted using ANN with SPSS 24.0 program. Data partitioning using a multi-layer perceptron (MLP) provided a training and verification ratio of 90:10, and hidden neurons in the design had sigmoid functions for both the hidden and result layers (no synaptic weights were used). Table 6 shows the analysis results of ANN conducted for the 4 groups of 2 × 2 cells (male-unmarried, male-married, female-unmarried, female-married). The results revealed that the impact of job and organizational engagement on creativity was the most important factor across all groups. In the case of male-married and female-unmarried groups, organizational engagement had the greatest importance on creativity, while in male-married and female-married groups, job engagement had the greatest importance on creativity. Among the sub-dimensions of intra entrepreneurship, CSR support was the most important in the female-unmarried, female-married, and male-unmarried group, respectively. On the other hand, innovativeness was the most important in the male-married group.

**Table 6:** ANN analysis: the importance of intra entrepreneurship, organizational engagement, and job engagement for creativity.

	**Male-unmarried** **(*****n*** **=** **69)**		**Male-married** **(*****n*** **=** **129)**		**Female-unmarried** **(*****n*** **=** **48)**		**Female-married** **(*****n*** **=** **39)**	
	**Importance**	**Normalized importance (%)**	**Importance**	**Normalized importance (%)**	**Importance**	**Normalized importance (%)**	**Importance**	**Normalized importance (%)**
Organizational engagement	0.359	95.2	0.242	74.7	0.323	83.3	0.358	81.1
Job engagement	0.377	100.0	0.324	100.0	0.387	100.0	0.441	100.0
Innovativeness	0.042	11.1	0.225	69.6	0.021	5.4	0.018	4.1
Pro-activeness	0.021	5.6	0.022	6.7	0.063	16.3	0.023	5.1
Risk-taking	0.055	14.5	0.089	27.6	0.002	0.5	0.047	10.6
CSR support	0.147	38.9	0.098	30.4	0.204	52.7	0.114	25.9

## Discussion and Implications

We integrated the resource-based theory (RBT), engagement theory, and stakeholder theory to hypothesize that four dimensions of intra entrepreneurship influence organizational and job engagement as well as indirectly employee creativity in SMEs context. However, we found two of the four sub dimensions of intra entrepreneurship improve organizational engagement, but they do not influence job engagement which can increase employee creativity. The findings show that SME employees engage more in the organization when they perceive that their SME owners implement innovativeness and CSR support activities. However, our results do not show that proactiveness and risk taking are as important for engagement. This study's findings support the theory that engagement as a mediator as job engagement plays a pivotal role in increasing employee creativity. In sum, current research revealed that employee work engagement can be viewed as a full mediator in the relationship between intra entrepreneurship and creativity. This research not only contributed to the current resource-based theory and engagement theory but also provided managerial implications for human resource management in the context of SMEs.

### Theoretical Implications

First, this study developed a research model that included CSR support factors in the measure of intra entrepreneurship and explained the effect on employee outcomes such as organizational and job engagement and employee creativity in the SME setting. Previous studies in the SME industry have been conducted in terms of three dimensions (innovativeness, pro-activeness, and risk-taking) of intra entrepreneurship. Based on stakeholder theory, this study proposed that CSR support needs to be included as part of entrepreneurship in which employees can engage in their organization and on their job. By investigating the proposed model, we analyzed the distinctly direct effects of four dimensions of intra entrepreneurship on organizational and job engagement as well as the indirect effects of entrepreneurship dimensions on employee creativity. Our findings that proactiveness sub dimension does not have a positive effect on engagement, suggests that as individuals adapt to the social environment at work, its importance is likely decreased for employees, especially if they are not challenged to do so (Farrukh et al., 2021). Second, originating in engagement theory, this study divided employee engagement into organization and job dimensions. This attempt helps SME owners/CEOs identify exactly how they will increase employee engagement at the organizational and individual level. In particular, the findings that innovativeness and CSR support affect organizational engagement and subsequently increase job engagement that increases creativity imply that employee engagement management should be differently at the organizational and individual level. Accordingly, this study confirmed why employee engagement should be managed in order to increase creativity. Third, based on the socialization process and social role theory, this study examined the roles of gender and marital status in the framework of intra entrepreneurship—employee engagement—creativity using ANN. The attempt of this study provides knowledge of the complex combinations of employees' socio-demographics that can predict and manage their attitudes and behaviors to explain how they impact organizational and job engagement of employees.

### Managerial Implications

Managerial implications can be expounded from this study. First, the findings show that employee engagement is an important driving force and determinant of organizational success in a fiercely competitive market as it improves employee creativity. Intra entrepreneurship is a tool to foster an entrepreneurial culture that promotes creativity and innovation (Kassa and Raju, 2015), as mediated by organizational engagement. Therefore, for CEOs and managers, hiring and retaining employees who are enthusiastic about their work is very important in inducing employee creativity and strengthening the company's competitiveness. From this point of view, the results of this study provide implications such that the relationship between the SME owners' entrepreneurship and the employee's behaviors must be built into a two-way relationship that can be exchanged (Saks, 2006) for employees to do creative work.

Second, this study shows that entrepreneurial innovativeness and perceived CSR support influence job engagement through the complete mediating role of employee organizational engagement. These results suggest that in Korea, which has traditionally emphasized organizational culture and organizational adaptation rather than individualism, entrepreneurial innovativeness and CSR support induces employees' sense of pride and belonging as members, arousing positive engagement for the organization, which in turn leads to individual's job engagement. Therefore, it is necessary for SME owners and HR managers to clearly present the owner's innovative image in the SME's mission statement and vision so that it can be well-disseminated to employees. And SME owners need to create a progressive and dynamic organizational climate that actively embraces and implements innovative ideas from employees who are valued organizational members. In addition, to enhance employee engagement, SME owners must first increase organizational trust using organizational support, organizational fairness, and brand image in accordance with the characteristics and orientation of the company. At the same time, SMEs should enhance employee loyalty through incentives based on organizational performance, such as for performance of departments and teams, rather than incentives based on individual performance. Meanwhile, when hiring new employees, companies should hire creative talent who sympathize with the company's vision and mission (Tian and Robertson, 2019) and include programs related to entrepreneurship when training new employees to boost employee engagement.

Third, this study shows that SME owners' CSR support has a direct and strong influence on employees' organizational engagement. These results mean that CSR is recognized as an essential element of management as well as innovativeness in SMEs. In addition, the results of sensitivity analysis showed that unmarried men recognize the importance of CSR more than married men. Even in the case of women, unmarried women recognize the importance of CSR more than married women. The findings support some studies showing that CSR support of SMEs owners enable employees feel of sense of organization, and as a result can enhance employees' organizational and job attitudes such as engagement (Abdelmotaleb et al., 2018) and performance such as creativity (Hur et al., 2018; Chaudhary and Akhouri, 2019). Therefore, the findings provide implications that the inclusion of unmarried employees in the members in charge of CSR related activities, such as internal and external customers, social contribution, environment, ethics, and laws, and spreading CSR programs through educational programs, intranets, and newsletters will be effective in enhancing creativity.

However, in this study, risk-taking did not affect employee engagement, which is consistent with the study of Kreiser et al. (2013). In general, employees working in SMEs recognize that SMEs have shorter survival periods and inferior salaries and welfare benefits than large companies. Therefore, the findings imply that when the owner of SMEs is risk-taking, employees feel anxiety about employment and show a tendency to avoid risk-oriented SMEs. In the case of men, the risk aversion tendency is more pronounced after marriage. Therefore, if the owner proposes a vision for the stable growth of the company, minimizes the burden on the consequences of taking risks, rewards for success, and encourages employees to take risks, which in turn can lead to employee creativity. Meanwhile, pro-activeness also did not influence employee engagement, suggesting that engaged employees need to be challenged and rewarded by SME owners to think proactively in the workplace. Also, the finding suggests that psychological capital of individuals which impacts their resilience to bounce back when ideas fail, may be mediating the relationship between risking taking and their full engagement, whereby employees invest their emotional and cognitive energies into their work (Bakker et al., 2011; Pandey et al., 2021).

### Limitations and Future Research

There are certain limitations to this study First, the data were collected from SMEs' employees in South Korea, and the sample size was relatively small. This could very well impact the generalization of research. Therefore, future studies need to increase the sample size. In addition, future studies need to collect and compare data from SME employees in different countries. Finally, future studies may enhance the research model by including other dimensions of entrepreneurship such as autonomy and competitiveness (REF) and other variables such as self-efficacy or employee's regulatory focus (promotion vs. prevention) to examine how entrepreneurship influences employee creativity.

## Data Availability Statement

The raw data supporting the conclusions of this article will be made available by the authors, without undue reservation.

## Author Contributions

TW-K and YK-L designed the study, collected the data, contributed to the manuscript writing, and data analysis. PN-S and CI-P contributed to the literature review, manuscript writing, and data analysis. All authors revised the manuscript critically and approved the final version of the manuscript.

## Funding

This research was supported by the Bisa Research Grant of Keimyung University in 2019.

## Conflict of Interest

The authors declare that the research was conducted in the absence of any commercial or financial relationships that could be construed as a potential conflict of interest.

## Publisher's Note

All claims expressed in this article are solely those of the authors and do not necessarily represent those of their affiliated organizations, or those of the publisher, the editors and the reviewers. Any product that may be evaluated in this article, or claim that may be made by its manufacturer, is not guaranteed or endorsed by the publisher.
